# Increasing Awareness of Proper Disposal of Unused and Expired Medication Using a Knowledge-Based Disposal Management System

**DOI:** 10.1155/2022/1797440

**Published:** 2022-02-28

**Authors:** Deemah Alshehri, Haneen Banjar

**Affiliations:** ^1^King Abdulaziz and His Companions Foundation for Giftedness and Creativity (Mawhiba), Riyadh, Saudi Arabia; ^2^Computer Science Department, Faculty of Computing and Information Technology, King Abdulaziz University, Jeddah, Saudi Arabia; ^3^Centre of Artificial Intelligence, Precision Medicines, King Abdulaziz University, Jeddah, Saudi Arabia

## Abstract

Medicines are used daily in Saudi Arabian homes. However, when these medicines are no longer needed, most people dispose of them incorrectly, forgetting the harmful impact of improper disposal. Inadequate awareness and knowledge are major reasons for improper disposal. In this study, we create a broad inclusive knowledge base that includes many types of medications available in Saudi homes and provides guidance on how to dispose of them as a means of raising awareness on correct disposal methods and preventing harmful impacts on both the environment and society. The study primarily aims to understand societal behaviour regarding the disposal of unused and expired medications and develop a prototype of a knowledge-based system that helps raise awareness of correct disposal methods for unused and expired medications. The data in the knowledge base are presented in tables that are easy to understand and comprehend, and the recommendations are also easy to apply and practice in everyday life. The results from the survey show that 66.8% of the 310 participants had unneeded medications in their homes, and only 14.9% knew how to dispose of unusable medications, while only 6.5% knew how to dispose of expired medications. Overall, the research studied Saudi society's behaviour regarding unused and expired medications, and we created a prototype of a knowledge-based system designed to increase awareness of proper disposal and management of unused and expired medications.

## 1. Introduction

Medications are chemical substances introduced into the body to cure a disease or pathological condition, relieve the symptoms of a specific illness, or simply to prevent disease [1]. Although medications play a considerable role in our daily lives, advancements in the medical field have contributed significantly to a remarkable increase in drug waste. This is attributable to the growing number of patients and overprescription by health care providers. The ensuing drug and medication waste has resulted in biological maladies and ethical challenges, and it has a negative impact on the environment [2, 3]. It is important to raise awareness about how to dispose of expired medications correctly and what to do with unused medications.

Most homes in Saudi Arabia have medicine cabinets filled with medicines, some of which have expired, and others that are not needed. Furthermore, most people dispose of medications incorrectly. For example, some people pour liquid medicines into toilets or bathtubs, which could cause pollution to our environment, especially by poisoning the waterways, which harms marine life, negatively affects humans and animals, and may spawn new diseases with a potentially dire impact on Saudi Arabian society [4]. There are also people who dispose of drugs by throwing them in the trash (e.g., garbage cans), which also increases the hazard potential of these chemicals when they are exposed to the sun or eaten by animals, causing harm [2]. Even when medicines are not disposed of in any of these ways, keeping unneeded medications at home is extremely unwise because it could lead to accidental poisoning. When old drugs are not disposed of correctly and are instead kept in wardrobes or freezers, they are easily within reach of kids and pets, who may ingest them, leading to poisoning. In terms of economic impact, medicine wastage constitutes a large loss of financial resources expended by Saudi Arabia on the provision of healthcare for its citizens [5]. Consequently, this research proposes a technical solution that addresses these problems. First, we examine Saudi Arabia's societal behaviour regarding unused and expired medications and then explore an effective solution for increasing awareness to address this issue.

Many studies have investigated the level of knowledge on medication waste in different countries. In Saudi Arabia, a study in Riyadh showed that approximately 65% of the respondents keep medicines until they expire, 48.1% throw medicines in the garbage at home, and only 5.4% return medicines to medical stores [6]. Another study [7], conducted in Jeddah, reported that 91.57% of the respondents disposed of their expired medications by discarding them with household waste, while only 2.98% returned expired medicines to medical stores. Regarding unused medications, however, 67.07% of the respondents disposed of them with household waste, and 10.84% donated them. In Kuwait [8], the numbers are different, with the most common method of medication disposal among respondents being to throw them in the garbage, as reported by 76.5% of the respondents. Flushing medication down the toilet was the method of choice for 11.2% of the respondents in the Kuwait study. In India, a survey of 393 respondents (32% male and 68% female) discovered that the most common disposal method for old or unused medicines was as household trash (67%). Approximately 353 people (about 90%) knew how bad it was to dump chemicals improperly. However, it was observed that the participants had only partial knowledge of proper drug disposal, and a lack of practice of safe disposal methods was observed among 264 respondents (67.1% of the total sample) [7]. Another study [9], conducted in Delhi, the national capital territory of India, reports that the majority of consumers (89.9%) believe medicine expiry dates indicate that the medicine has expired, has begun producing toxic chemicals, and has diminished or no medicinal benefits. Nearly nine out of ten (87%) consumers keep medicines at home, and over 90% discarded expired medications after keeping them for a few days past the expiry date. After the expiration date, 73% of consumers primarily tossed out their expired medicines with household trash. A study conducted in Indonesia [10] reported that nearly 95% of the participants kept unused medicines in their homes. The most popular method of disposing of old medicines was dumping them in household garbage, as reported by more than 80% of the study participants. About 80% of the participants were unaware of proper medicine disposal techniques, and more than 50% were unaware that unsafe medicine disposal practices could harm the environment and negatively impact public health. In addition, in Palestine, Al-Khatib et al. [3] measured and assessed current management practices at three hospitals in Jenin's district that produce medical waste. The total general and hazardous waste generation rates in all hospitals surveyed were 0.97 kg/bed/day and 0.78 kg/bed/day, on average. Although in our study the focus is on expired and unused medication at home, Al-Khatib et al. recommended, after their significant results, that cooperation among many government agencies and nongovernmental organizations working in this subject is important. In addition, health care institutions should be required to establish a medical waste management system that is safe and hygienic for workers, the public, and the environment with a minimum amount of risk to these groups. Finally, they recommended occupational health and safety training as an essential part of protecting healthcare workers and waste handlers from potential harm. Many of these studies are limited to surveys that assess public awareness of medicine disposal, and there is no previous study that has considered creating a prototype of a knowledge-based system that will help guide people to dispose of medicine correctly.

Regarding the proposed solution, knowledge-based systems are designed to ensure highly accurate decision-making or recommendations by effectively and appropriately using data, information, and knowledge management for convergence industries. These systems are based on artificial intelligence and use information and communication technologies to aid decision-making and make recommendations [11]. The extraction of knowledge from sources of expertise is the most essential factor in knowledge-based systems, and this knowledge is then transferred to a knowledge base and an inference engine. Knowledge is an assortment of details, procedures, and opinions typically presented as tables, rules, charts, or graphs [12]. Using a knowledge-based system can facilitate an increase in awareness about proper medication disposal. Similarly, to ensure scientific homogeneity and accountability in the healthcare sector, more tools and methods should be introduced into regular clinical practice to help clinicians make more consistent treatment decisions. An overall reduction in cost, improved quality of care, greater patient satisfaction, and favourable public opinion are among the benefits to be expected from the creation of a smart clinical information management system that functions like a knowledge base for the healthcare system, in which both clinical information and knowledge can be collated and accessed [13]. In this study, the information was sourced from the World Health Organization guidelines [14]. This study aims to raise awareness about proper medication disposal practices and to educate consumers who are uneducated about this issue by creating a prototype of a knowledge-based system that increases awareness of the impact of improperly disposed medicines and ensures scientific homogeneity with the medicine disposal guide developed by the World Health Organization. The objectives are as follows: (i) to understand societal behaviour regarding the disposal of unused and expired medications and to sample opinions on a support system that increases awareness about medicine disposal; (ii) to collect information from government documents to create a knowledge base on how to properly dispose of expired and unused medications; and (iii) to develop a prototype that automatically guides the consumer on correct disposal methods for unused and expired medications.

## 2. Methods

The framework of this study is divided into two phases: a survey to understand societal behaviour, which uses descriptive analysis to explain societal behaviour and needs, and a second phase involving building a prototype for a knowledge-based system.

### 2.1. Phase I: Understanding Societal Behaviour

**Figure 1:**
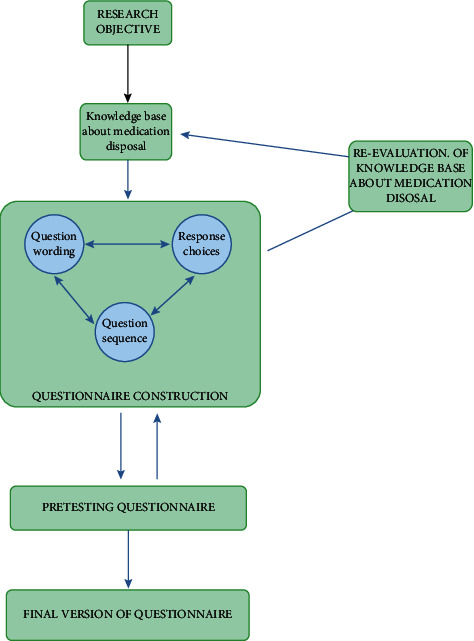
Questionnaire creation process and the points considered during the process.

To gain a better understanding of existing medication disposal procedures and to determine what people think about a knowledge-based system for raising awareness regarding proper medication disposal. The questionnaire method is used for gathering data and information. The questionnaire framework organizes the process and helps detect the objectives and main points, as shown in Figure 1. Firstly, we aim to understand societal behaviour regarding the disposal of unused and expired medications and to sample opinions on a support system that increases awareness about medicine disposal. The outcome from the questionnaire should assist in building the knowledge base. The wording of the questions, question sequence, and response choices were evaluated to construct a suitable questionnaire. Then, the questionnaire was pretested before use, and the required verification of the final version was performed.

The questionnaire language was developed in Arabic and pretested on qualified respondents. To create the questionnaire, the Google Forms platform was used because of its ease of use for gathering and analysing data and because it is familiar to the target audience of respondents, ensuring a hitch-free survey. During the development of the questionnaire, some changes were made after the pretesting process, with open-ended questions rephrased to elicit close-ended responses to limit the length of responses. Furthermore, an additional response option labelled “other” was added to allow respondents to record their response if it was different from the response choices provided. Respondents were informed about the objective of the research while filling out the questionnaire. Sociodemographic data were extracted from the questionnaire, including why the respondents had spare medicines at home (to determine if lack of knowledge about the correct disposal was the reason), their opinion on the best way to dispose of medications (the purpose of this question was to find the best way to dispose of medications), and if they support the existence of a prototype that will help guide people on proper disposal of medication (this question was asked to assess whether there will be a supportive audience when the prototype is completed). We also asked whether the respondents think improper disposal of medication is an issue that should be tackled (to consider their perspectives on the issue). The questionnaire was distributed via social media, and the survey was conducted from June 2021 to July 2021. The data was analysed using a basic comparison of the responses. In some cases, there were unanswered questions, which were treated as missing values, and only fully answered questions were used to calculate the results.

### 2.2. Phase II: Knowledge-Based System

**Figure 2:**
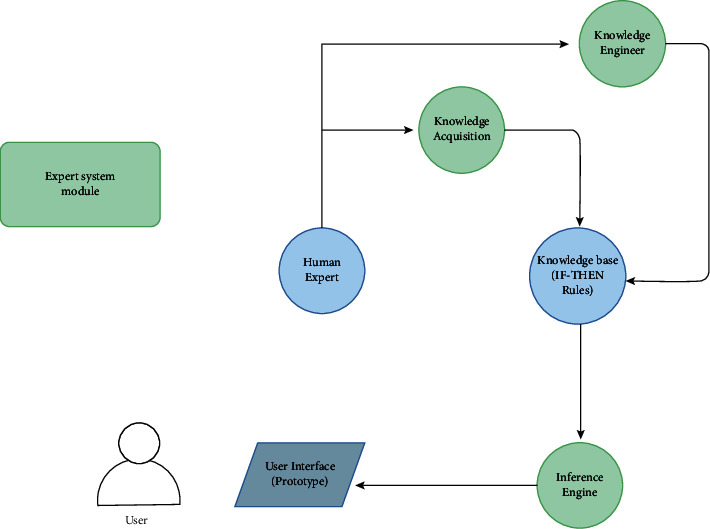
Framework of the knowledge-based system and its components.

A knowledge-based system involves knowledge acquisition, knowledge verification, an inference engine, a knowledge engineer, and an interface (Figure 2). First, for knowledge acquisition, all methods of medicine disposal in our system were sourced from the World Health Organization guidelines [15]. These methods were organized using IF-THEN rules and then sorted into tables according to the material, medication type, quantity, toxicity, and availability of sewers for liquid medications. There was also an exclusion criterion based on the following: (i) particularly difficult disposal methods that could cause danger if performed in homes, and (ii) the need for experts and specialists because the disposal methods are too advanced and cannot be implemented at home by the average person. During the process of creating the knowledge base, some information was filtered to limit the scope to the challenges facing Saudi Arabia alone, which involves finding proper disposal methods for unused and expired medications that can be safely implemented at home. However, useable medications that are no longer needed can always be donated to donation centres or hospitals. Second, regarding knowledge verification, all knowledge shared in our system was approved and validated by a pharmaceutical expert and then presented in tables. Third, the inference engine proved to be a crucial element in our system. After collating information using IF-THEN rules, the inference engine applies these rules from the knowledge base to each case to present a recommendation on the best approach for disposal. Fourth is the knowledge engineer component. This is an individual who develops advanced logic in computer systems to simulate human decision-making. Finally, after creating the knowledge-based system, a prototype was developed to represent the interface of the system. This interface guides users to proper medication disposal methods based on the medication type and provides directions to medication donation centres.

## 3. Results

The research results are divided into two sections, one for each phase of the study. The first section presents the survey findings on societal behaviour regarding the disposal of unused and expired medications and the surveyed opinions on a system to increase awareness of medicine disposal. The second section is on the development phase of the knowledge-based system, involving the collation of the information used to create a knowledge base on how to dispose of expired and unused medications, which is required to develop a prototype that automatically guides consumers and consequently raises awareness about proper disposal methods for unused and expired medications.

### 3.1. Phase I Results

**Figure 3:**
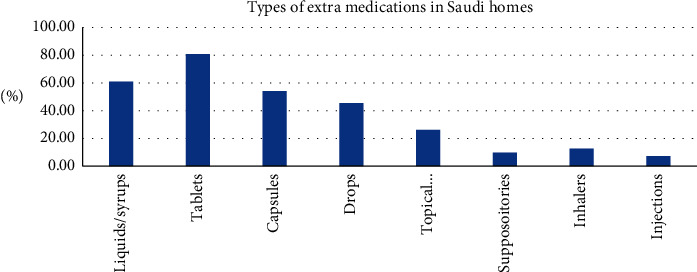
Types of surplus medications in Saudi homes based on the survey results.

Three hundred and ten respondents were surveyed. About two-thirds (66.8%) of the respondents reported that they have extra and unneeded medications at home. Respondents also indicated the types of medication they had at home (Figure 3), of which 60.9% were syrups or liquids, 80.7% were tablets, 54.1% were capsules, 45.4% were drops, 26.1% were topical medicines, 9.7% were suppositories, 12.6% were inhalers, and 7.2% were injections. This information was taken into consideration in the collation of knowledge during the second phase of the research.

Participants were also asked whether the medications they had at home were expired or useable, and 22.2% indicated that their spare drugs had expired. The next question enquired about the reason for keeping expired drugs, and 17.4% of the respondents indicated that they did not need the drugs. More than half of the respondents (63%) indicated that the treatment period had ended, 6.5% indicated that the individual for whom the drugs were prescribed had passed away, and 2.2% reported that the medications were an overprescription by healthcare provider. Sixty-three percent of the respondents agreed that improper disposal of expired medications is a problem that should be urgently solved. Regarding disposal methods, 93.5% of the respondents indicated that they were not aware of proper methods to dispose of expired medications, 91.3% threw expired medications in household garbage, 4.35% donated medications to charities or returned them to hospital, and 4.35% did not know how to dispose of medications. However, 80.4% of the respondents are supportive of a prototype application that would guide them on the proper disposal of expired medications.

**Figure 4:**
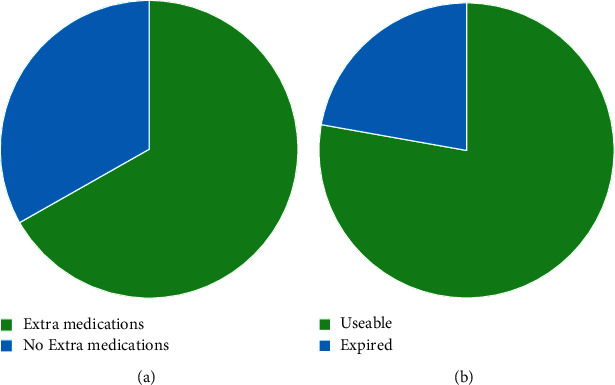
(a) Results from the questionnaire on how many Saudis have surplus medication at home. (b) Results from the questionnaire on the status of surplus medications in Saudi homes.

About 77.8% of the respondents found that they have surplus useable drugs at home. Although there were various reasons why they kept useable unneeded drugs, the main reasons were that the drugs were left over after their treatment period had ended (55.9%), there was a large quantity available (28%), and 8.1% said they did not need them. Like the responses on expired medications, 78.3% of the respondents agreed that improper disposal of useable medications is a problem that should be solved urgently. Only 14.9% of the respondents indicated that they knew how to dispose of useable medications, while the remaining 85.1% indicated that they were unaware. There were many methods employed in disposing of useable unneeded medications, but most of the respondents indicated that they threw out the medications with household garbage, while only 24.22% thought of donating these drugs. Overall, 80.7% of the respondents indicated that they support the availability of donation centres, and 91.9% indicated that they support the existence of a prototype application that would guide them on the proper disposal of medications (Figure 4).

Respondents who did not have any spare drugs at home were also asked if they thought improper medication disposal was an issue that should be tackled, and 55.3% of these respondents agreed. They were also asked whether they were aware of the proper methods of medication disposal, and 84.5% indicated that they did not have any knowledge of proper medication disposal. Finally, 74.8% support the availability of donation centres, and 92.2% are in support of the existence of a prototype application that guides them on the proper disposal of medication.

### 3.2. Phase II: Development of the Knowledge-Based System

This section presents the construction of the components of the proposed knowledge-based system. There were exclusion criteria because (i) there were particularly difficult disposal methods that posed some danger if performed in a home, and (2) some methods of disposal required experts and specialists because the methods were too advanced and cannot be implemented by the average person at home. Recommended disposal methods are summarized in Table 1 according to the sorting category [15]: solids, semisolids and powders, liquids, ampoules, anti-infective drugs, controlled substances, antineoplastics, disinfectants, and aerosol canisters. This information was sourced from the WHO guidelines.

**Figure 5:**
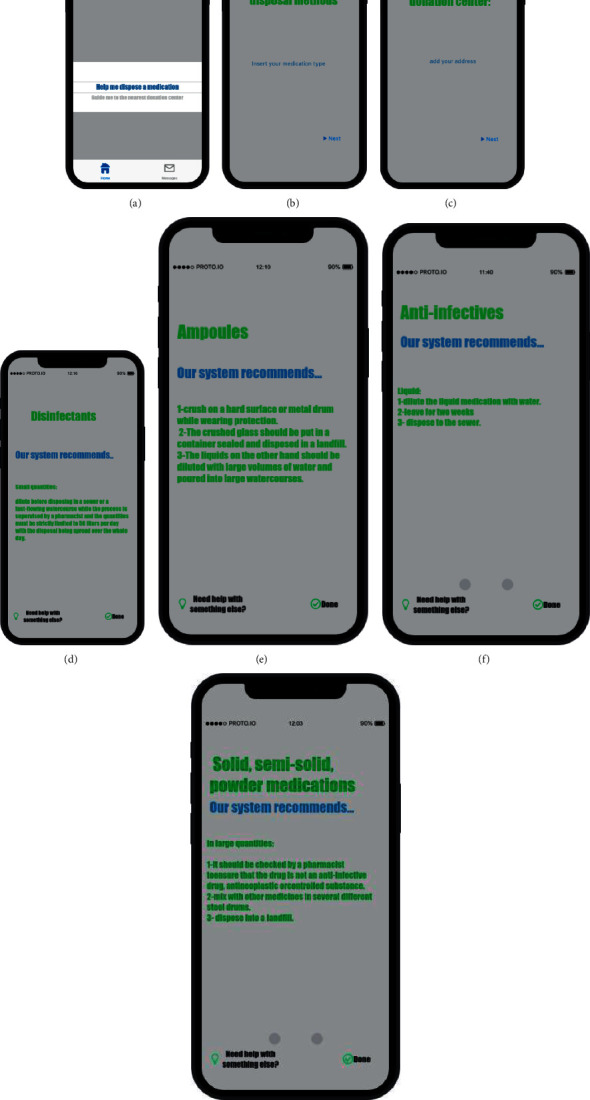
Prototype of a knowledge-based system for increasing awareness about the proper disposal of unused and expired medication: (a) home screen, (b) screen when the user requests guidance on the proper disposal methods, (c) screen when the user enters aerosol canisters or inhalers as the medication category for which they need guidance on disposal methods, showing the recommendation that appears, (d) screen when the user enters disinfectants as the medication category for which they need guidance on disposal methods, showing the recommendation that appears, (e) screen when the user enters ampoules as the medication category for which they need guidance on disposal methods, showing the recommendation that appears, (f) screen when the user enters anti-infectives in liquid form as the medication category for which they need guidance on disposal methods, showing the recommendation that appears, and (g) screen when the user enters solid, semisolid, and powder forms of medications in large quantities as the medication category for which they need guidance on disposal methods, showing the recommendation that appears.

In our knowledge-based system, the collated information is used to generate recommendations after a user selects the type of medication. The steps and procedures are presented in Figure 5. The interface for the prototype was designed on the Proto.io website.

## 4. Discussion

Disposal of medications is a critical problem confronting both healthcare providers and patients alike. Educating patients on proper drug disposal is a fitting role for pharmacists because it is a role that they are in a unique position to perform adequately. Safe disposal of medicines can have a significant positive impact on public health and the environment if patients are properly counselled on how to go about it properly. Disposing of medications properly and with care can help reduce the environmental burden that drugs place on the environment [16]. The first phase of the research proved that there is insufficient awareness about medication disposal and inadequate knowledge of the dangers posed by incorrect medication disposal in Saudi society. The findings of this study are similar to those of comparable studies [17, 18, 2].

It is essential to promote education as a means of raising awareness of the negative consequences of inappropriate medicine disposal. However, even among those who are aware of the dangers of inappropriate medication disposal, previous research showed that effective disposal methods are still lacking [2, 19]. Increased education on proper medicine disposal, particularly among college students, clearly has to occur [20]. In addition, there is also a need to increase awareness about medical disposal through training for nurses, doctors, and technicians in healthcare centres, medical laboratories, private clinics, dental clinics, and hospitals, as recommended in this study [21].

The second phase of the research created an inclusive knowledge-based system, which is intended to raise awareness and spread knowledge about the proper methods of medication disposal and presents the disposal methods in tables categorized according to medication type, quantity, toxicity, and availability of sewers for disposal of liquid medications. The results from the first phase of the research (survey) facilitated the selection of medication types focused on in the second phase. This made the research highly precise and thus highly utilitarian for Saudi society, although we studied all medication types and ended up with an inclusive knowledge base. The prototype developed works such that it increases knowledge about proper medicine disposal methods in Saudi society by using tables that present information extracted from the WHO guidelines. The tables summarize each medication type and its proper disposal methods. The tables are easy to understand, comprehend, and user-friendly. Hence, complexity is not an impediment to using the knowledge-based system developed in this study.

## 5. Conclusion

Through the development of a prototype knowledge-based system designed to raise consumer awareness of the consequences of improperly disposed of medicines and to ensure scientific homogeneity with the WHO medicine disposal guide, this research aims to increase consumer awareness and provide education on the subject for consumers who are not already knowledgeable. This study reveals that there is a need for more awareness of the proper disposal of medications in Saudi society and puts forward a prototype system that guides people on the proper disposal methods for expired and unused medications. There are several limitations to this research. First, the study was restricted to Saudi citizens. Future studies should assess whether our findings can be extrapolated to a broader population. The development of the proposed knowledge-based system will be the next step. Education through a knowledge-based system can raise awareness of correct disposal standards, which are essential for reducing drug waste.

## Figures and Tables

**Table 1:** Summary of disposal methods based on the type of pharmaceutical.

Type of Pharmaceutical	Disposal Method
Ampoules	1. Crush on a solid surface or metal drum while wearing protection.
	2. The crushed glass should be placed in a container, which should be sealed and disposed of in a landfill.
	3. Liquids, on the other hand, should be diluted in a large volume of water and poured into large water courses.
Disinfectants (small quantities)	Dilute before disposing of in a sewer or fast-flowing watercourse. The process should be supervised by a pharmacist, and the amount disposed must strictly be limited to fifty litters per day, with the disposal being spread over the entire day.
Aerosol, canisters, and inhalers	Dispose of in a landfill, scatter around the municipal solid wastes.
*Liquids*	

Sewers available	1. The medication to be disposed of should be inspected by a pharmacist to confirm that the drug is not an anti-infective drug, an antineoplastic, or a controlled substance.
	2. Flush into sewers.

No sewers available	1. Dilute the liquid medication in a large volume of water.
	2. Pour into large watercourses.
	OR
	1. Pour the liquid into a solid block within a plastic container or steel drum.
	2. Fill the block to 75% capacity with the liquid medicine.
	3. Fill in the remaining space by pouring in cement/lime mixture, plastic foam, or bituminous sand.
	4. Seal the drum.
	5. Place the drum at the base of a landfill.
	6. Cover the drum with municipal solid waste.
Liquid with little or no toxicity	Flush into a sewer.
Solid, semisolid, or powder	

In small quantities	1. Remove the outer packaging.
	2. Place in clean plastic containers or steel drums.
	3. Dispose of in a landfill.
	4. Immediately cover with municipal waste.

In large quantities	1. The medicines should be inspected by a pharmacist to ensure that the drug is not an anti-infective drug, an antineoplastic, or a controlled substance.
	2. Mix with other medicines in several steel drums.
	3. Dispose of in a landfill.
*Solid*	

Antineoplastics	1. Separate the medicine from other medications.
	2. Ensure that the container is safely sealed.
	3. Return the medicine to your supplier for proper disposal.
	OR
	1. Place it in a steel drum filled 50% with drugs.
	2. Add a well-stirred mixture of cement, lime, and water in the proportion of 15: 15: 5 (by weight) until the drum is full.
	3. Seal the drum.
	4. Leave it to sit for 7–28 days (this should form a firm, solid block).
	5. Dispose of in a landfill.

Controlled substance	1. Remove the outer packaging.
	2. Place in clean plastic containers or steel drums.
	3. Dispose of in a landfill.
	4. Cover with municipal waste. This step should be supervised in keeping with local regulations.
	OR
	Incinerate

Anti-infectives	1. Place the medication in a solid block within a plastic container or steel drum.
	2. Fill the block to 75% capacity with the solid medicine.
	3. Fill in the remaining space by pouring in cement, cement/lime mixture, plastic foam, or bituminous sand.
	4. Seal the drum.
	5. Place the drum at the base of a landfill.
	6. Cover the drum with municipal solid waste.
	OR
	Incinerate
*Liquid*	

Anti-infectives	1. Dilute the liquid medication in water.
	2. Leave for two weeks.
	3. Dispose of in a sewer.
Ampoules	

Antineoplastic	1. Place the liquid/solid in a solid block within a plastic container or steel drum.
	2. Fill the block to 75% capacity with the liquid/solid medicine.
	3. Fill the remaining space by pouring in cement, cement/lime mixture, plastic foam, or bituminous sand.
	4. Seal the drum.
	5. Place the drum at the base of a landfill.
	6. Cover the drum with municipal solid waste.

## Data Availability

The data used to support the findings of this study are available from the corresponding author upon request.
